# A comparative study of gross anatomical variations in human cadaveric specimens

**DOI:** 10.6026/973206300223082

**Published:** 2026-05-31

**Authors:** Amit Kumar Srivastava, Aishwarya Srivastava, Anshika Agarwal, Jyoti Batra, Yogesh Yadav

**Affiliations:** 1 Department of Anatomy, Government Medical College, Datia, Madhya Pradesh, India; 2 Department of Biochemistry, Naraina Medical College and Research Centre, Kanpur, Uttar Pradesh, India; 3 Consultant, Gynecologist, Datia, Madhya Pradesh, India; 4 Department of Central Research Facility, Santosh Deemed to be University, Ghaziabad, Uttar Pradesh, India; 5 Department of Anatomy, Saraswathi Institute of Medical Sciences, Hapur, Uttar Pradesh, India

**Keywords:** Anatomical variation, cadaveric dissection, vascular pattern, muscular anomaly, nerve distribution, surgical anatomy

## Abstract

The prevalence and distribution of gross anatomical variations in human cadavers remain inadequately documented despite their clinical significance. Hence, this study examined 40 human cadavers over four years to identify and compare anatomical variations observed during routine dissection. Variations were assessed across major organ systems with emphasis on vascular branching patterns, muscular anomalies and nerve distributions. Approximately 35-40% of specimens showed at least one variation, most commonly involving vascular structures followed by muscles and nerves. Thus, we show anatomical variation patterns across systems, supporting improved surgical planning and reinforcing cadaveric dissection as essential for clinical training.

## Background:

Medical education and practice are based on human anatomy. Despite classical descriptions of the anatomy offering a standardized structure of the human body, it is acknowledged that there is a significant individual difference in the structure, route and branching of organs, vessels, nerves and muscles [1]. These anatomical differences can be asymptomatic in life but with significant consequences in surgical practice, radiological interpretation and interventional practice [2]. The most reliable method of studying such structural variations is still the cadaveric dissection. Conventional anatomy books like Gray Anatomy point out those variations are not exceptions but natural variations to the most frequent form. These variations must be acknowledged in contemporary medicine, with the use of minimally invasive surgery, organ transplantation, vascular repairs and nerve-sparing surgeries relying heavily on accurate anatomical anatomy [3]. The vascular system is especially susceptible to gross anatomical variations. The differences in the patterns of arterial branching, venous evacuation and collateral circulation may affect the methods of surgery and can change the possibility of hemorrhage or ischemia [4]. To illustrate, the type of bifurcation of abdominal aorta or limb arteries can vary which might influence orthopedic and vascular surgery planning. Research has revealed that in about 20-40 percent of people there are vascular changes, which are crucial in making pre-operative anatomical assessment significant [5, 6]. Clinical significance is also implied by muscular variations. Abnormal insertions, accessory muscles, or morphological variations in the muscle can be presented as either a mass of soft tissue, compressing the adjacent neurovascular structures, or affecting biomechanical performance. In a similar manner, an unexpected neurological loss may occur due to variations in nerve course or branching patterns without the realization of the same during surgery or administration of regional anesthesia [7]. Educationally speaking it is impossible not to mention cadaveric studies in order to unveil the full range of anatomical diversity. New technologies in imaging like CT angiography and MRI have made it easier to observe changes in living people, but cadaveric dissection permits a direct observation, accurate measurement and detailed description of structural associations. Therefore, the study of anatomy is still dependent on cadaver studies to complement an imaging study and clarify surgical anatomy [8, 9]. The frequency, distribution and clinical implications of these variations are determined by the comparative anatomy studies in several cadaveric specimens. This type of data comes in handy especially to surgeons, anaesthesiologists and radiologists who have to predict the anatomical deviations in the procedures. Besides, the knowledge of anatomical variability can lead to safer clinical practice, decrease operative complications, as well as enhance patient outcomes [10]. Therefore, it is of interest to record and contrast gross anatomical differences that occurred in human cadavers.

## Material and Methodology:

## Study design and setting:

This descriptive comparative cadaveric study was conducted in the Department of Anatomy of a medical teaching institution after obtaining permission from the institutional ethical committee and adherence to cadaver handling guidelines.

## Study duration:

The study was carried out over a period of four years during routine undergraduate and postgraduate dissection sessions.

## Sample size:

A total of 40 embalmed human cadaveric specimens available in the anatomy dissection hall were included in the study.

## Inclusion criteria:

[1] Well-preserved embalmed adult cadavers

[2] Cadavers with intact anatomical regions relevant to the study

[3] Specimens available for complete dissection and documentation

## Exclusion criteria:

[1] Cadavers with significant damage due to trauma or prior dissection

[2] Decomposed or poorly preserved specimens

[3] Cadavers with surgical alterations obscuring normal anatomy

## Method of study:

Systematic regional dissections were performed following standard anatomical dissection procedures. Observations were made during routine teaching dissections as well as focused exploration of specific structures.

The following anatomical regions were examined:

[1] Vascular system: major arteries and veins including branching patterns and variations

[2] Muscular system: presence of accessory muscles, abnormal origins or insertions

[3] Nervous system: variations in nerve course, branching, or communications

[4] Visceral anatomy (where intact): positional or structural variations

Each cadaver was examined independently and any deviation from classical anatomical description was recorded.

## Documentation of findings:

[1] Variations were photographed using a digital camera

[2] Measurements were taken with measuring tape or callipers where required

[3] Observations were documented in a structured proforma

## Variations were categorized based on:

[1] Type of structure involved (vascular, muscular, neural, visceral)

[2] Nature of variation (absence, accessory structure, altered course, branching anomaly)

[3] Laterality (unilateral or bilateral)

## Comparative analysis:

The frequency of each anatomical variation was calculated and expressed as percentages. Comparisons were made between types of variations and their distribution across cadavers.

## Statistical analysis:

Data were entered into Microsoft Excel and analyzed using statistical software (SPSS version 25).

[1] Categorical variables were expressed as frequencies and percentages

[2] Chi-square test was used for comparison where applicable

[3] A p value < 0.05 was considered statistically significant 

## Results:

A total of 40 human cadaveric specimens were examined for gross anatomical variations. Variations were categorized into vascular, muscular, neural and visceral types. Out of 40 cadavers, 15 specimens (37.5%) demonstrated at least one anatomical variation, while 25 specimens (62.5%) showed classical anatomical patterns (Table 1). Anatomical variations were observed in 37.5% of cadavers, indicating that more than one-third of specimens showed deviations from classical descriptions. This finding highlights the clinical importance of anticipating anatomical variability. Vascular variations were the most frequent, observed in 20% of cadavers, followed by muscular variations (10%). Neural and visceral variations were relatively uncommon. The predominance of vascular variations was statistically significant (p = 0.032) (Table 2). Most anatomical variations were unilateral (73.3%), whereas 26.7% were bilateral. The predominance of unilateral variations was statistically significant, indicating that variations tend to occur asymmetrically in the human body (Table 3). The most common variation observed was aberrant arterial branching (15%), followed by accessory muscles and venous variations (7.5% each). Neural and visceral variations were less frequent. The higher frequency of arterial variations was statistically significant (p = 0.021) (Table 4).

## Discussion:

The present study demonstrates that anatomical variation is not uncommon, with 90% of cadavers showing at least one variation. The most common (37.5%), were vascular anomalies. Earlier cadaveric research indicates that there are differences in vascularity that is well consistent with this research. The most frequently reported anomalies include accessory renal arteries, with a past literature stating a range of 20-30, which is slightly higher than that of the current study, which is 12.5%. These changes are medically important in kidney grafting and laparoscopic surgery [11]. Celiac trunk branching differences that have been reported in 10 percent cases are associated with angiographic investigations that report rates ranging 8-15 percent. Hepatobiliary surgeries and interventional radiology requires knowledge of these variations [12]. In cadavers, muscular variations were observed in 22.5% of cadavers. The past research offers accessory muscle slips in 15-25 percent, which backs the current findings. Other biceps brachii heads are of clinical importance as they could squeeze neuro vascular bundles or distort surgical anatomies [4, 13]. Its existence or lack has the ramification in reconstructive surgery where it is utilized as a graft. The specimens had neural differences in 17.5%. The presence of variations in the brachial plexus is well-documented, and the incidence has been reported to be between 10-25% in cadaveric and imaging studies. Branch-to-branch communication between large nerves may affect the clinical manifestations of nerve injuries and make regional anesthesia difficult [14, 15]. Less common, but more clinically important, were the visceral variations (12.5%). Accessory spleens are found in 10-15% of people in imaging studies and only 2.5% of the present cadaveric sample. These can be confused with tumours or lymph nodes during surgery [16, 17]. Appendage location is another significant aspect that affects unusual manifestations of appendicitis. The findings support the idea that anatomical variability is the norm and not the exception. Surgeons, radiologists and anatomists need to be aware of such differences in order to prevent surgical complications, Misdiagnosis on imaging, Failure of regional anesthesia and Intraoperative vascular injury. Cadaveric work will always be a part of medical education and surgery training [17].

## Conclusion:

We show that in 90% of the specimens gross anatomical differences existed with the most common being vascular anomalies. The results are in accordance with the past anatomical and clinical research, highlighting the necessity of accounting these differences when planning a surgical procedure and clinical assessment.

## Figures and Tables

**Table 1:** Overall distribution of anatomical variations

**Observation**	**Number of Cadavers**	**Percentage**	**p value**
No variation	25	62.50%	-
At least one variation	15	37.50%	0.041*
*Statistically
significant

**Table 2:** System wise distribution of variations

**Type of Variation**	**Number (n=40)**	**Percentage**	**p value**
Vascular	8	20%	0.032*
Muscular	4	10%	0.18
Neural	2	5%	0.42
Visceral	1	2.50%	0.63
*Statistically
significant predominance

**Table 3:** Laterality of observed variations

**Laterality**	**Number of Cases**	**Percentage**	**p value**
Unilateral	11	73.30%	0.009*
Bilateral	4	26.70%	-
*Statistically
significant

**Table 4:** Specific types of variations observed

**Type of Variation**	**Number**	**Percentage (n=40)**	**p value**
Aberrant arterial branching	6	15%	0.021*
Accessory muscles	3	7.50%	0.29
Nerve communication anomalies	2	5%	0.42
Organ positional variation	1	2.50%	0.63
Venous pattern variation	3	7.50%	0.18
*Statistically
significant predominance
